# The General Practitioner and the New Health Bill

**Published:** 1920-07-03

**Authors:** 


### THE GENERAL PRACTITIONER AND THE NEW HEALTH BILL.

FROM A CORRESPONDENT.

In a recent issue we wrote in general terms of the value of the "New Hospitals" which may be proposed in the Health Bill which Dr. Addison is understood to be drafting. Here let us deal more particularly with the advantages that should accrue to the general practitioners and through them to the science of medicine itself from the contemplated linking up of the medical services of the country.

Sir James Mackenzie has expressed the opinion that general practitioners should be research workers, but while his own career forbids us to regard this as impossible in given instances, it is certain that under existing conditions no such desirable end could ever be attained on anything like a general scale.

The general practitioner has laboured hitherto under crippling disadvantages in his work; indeed it is not too much to say that he has been handicapped from the very commencement of a training which has brought him, only accidentally, into contact with those manifestations of disordered health which constitute so large a proportion of his work in after life. The wards of his hospital contained only cases of serious disease, and yet at least 80 per cent. of his patients in practice are suffering merely from some slight ailments which disturb their general health. For these they need and seek advice. A patient comes to the young medico complaining of various abdominal symptoms. In hospital he had seen many such cases, but they were cancer, gastric or duodenal ulcer, appendicitis, etc. At once a list of these possible troubles flashes through the doctor's mind, and he sets to work diligently to run the offender to earth. In an overwhelming majority of cases he draws a blank, which is satisfactory. What is not so satisfactory is the fact that while he would know how to treat a gastric ulcer, he does not know what to do with a case of "indigestion." He has, perhaps, seen such a case in student or resident appointment days, sent in as query this, that, or the other. Everyone concerned, from the senior members of the staff down to his own humble self, had been keenly interested in the patient until exhaustive examination had failed to reveal any organic disease, whereupon enthusiasm had immediately evaporated and the patient had been sent back to home and doctor. In his present quandary he recalls this case, and inwardly blesses the "teachers" who finished his instruction where properly it should have commenced.

But one day a patient with the old familiar list of symptoms arrives, and examination reveals an unmistakeable "lump." "Dr. X. ordered the patient's removal to hospital." Thus robbed by social and economic conditions of all further participation in a case whose proper treatment he has spent years of labour to master, he is expected, nevertheless, to keep up to date.

Medicine is at the dawn of a new era. The air is instinct with change. Instruments both for diagnosis and treatment are becoming multiplied on every hand. Even the great clinical physicians are now considered inadequate to the demands of increasing knowledge, and "team work" is the cry of the hour. Clinician, bacteriologist, pathologist, physiologist, and $x$-ray expert, are to act in concert to a degree hitherto undreamed of. The problem is assuming serious proportions, and it would seem that the time is not far distant when even the millionaire will need to be treated in hospital if he is to receive the best results of modern science.

In view of the needs both of practitioner and patient there could be no more appropriate time than the present for the commencement of reform.

With facilities for keeping in touch with his more serious cases and participating in the unspeakable advantages of constant consultations and actual treatment, the general practitioner should become a different man.

